# Therapeutic potential of autophagy-enhancing agents in Parkinson’s disease

**DOI:** 10.1186/s13024-017-0154-3

**Published:** 2017-01-25

**Authors:** Tim E. Moors, Jeroen J. M. Hoozemans, Angela Ingrassia, Tommaso Beccari, Lucilla Parnetti, Marie-Christine Chartier-Harlin, Wilma D. J. van de Berg

**Affiliations:** 1 Department of Anatomy and Neurosciences, Section Clinical Neuroanatomy, Amsterdam Neuroscience, VU University Medical Center Amsterdam, Amsterdam, The Netherlands; 2 Department of Pathology, Amsterdam Neuroscience, VU University Medical Center Amsterdam, Amsterdam, The Netherlands; 3 Department of Pharmaceutical Sciences, University of Perugia, Perugia, Italy; 4 Department of Medicine, Section of Neurology, University of Perugia, Perugia, Italy; 5 UMR-S 1172—JPArc—Centre de Recherche Jean-Pierre AUBERT Neurosciences et Cancer, University of Lille, Lille, F-59000 France; 6 Inserm, UMR-S 1172, Team “Early stages of Parkinson’s disease”, F-59000 Lille, France

## Abstract

Converging evidence from genetic, pathological and experimental studies have increasingly suggested an important role for autophagy impairment in Parkinson’s Disease (PD). Genetic studies have identified mutations in genes encoding for components of the autophagy-lysosomal pathway (ALP), including glucosidase beta acid 1 (*GBA1*), that are associated with increased risk for developing PD. Observations in PD brain tissue suggest an aberrant regulation of autophagy associated with the aggregation of α-synuclein (α-syn). As autophagy is one of the main systems involved in the proteolytic degradation of α-syn, pharmacological enhancement of autophagy may be an attractive strategy to combat α-syn aggregation in PD. Here, we review the potential of autophagy enhancement as disease-modifying therapy in PD based on preclinical evidence. In particular, we provide an overview of the molecular regulation of autophagy and targets for pharmacological modulation within the ALP. In experimental models, beneficial effects on multiple pathological processes involved in PD, including α-syn aggregation, cell death, oxidative stress and mitochondrial dysfunction, have been demonstrated using the autophagy enhancers rapamycin and lithium. However, selectivity of these agents is limited, while upstream ALP signaling proteins are involved in many other pathways than autophagy. Broad stimulation of autophagy may therefore cause a wide spectrum of dose-dependent side-effects, suggesting that its clinical applicability is limited. However, recently developed agents selectively targeting core ALP components, including Transcription Factor EB (TFEB), lysosomes, GCase as well as chaperone-mediated autophagy regulators, exert more specific effects on molecular pathogenetic processes causing PD. To conclude, the targeted manipulation of downstream ALP components, rather than broad autophagy stimulation, may be an attractive strategy for the development of novel pharmacological therapies in PD. Further characterization of dysfunctional autophagy in different stages and molecular subtypes of PD in combination with the clinical translation of downstream autophagy regulation offers exciting new avenues for future drug development.

## Background

The presence of Lewy bodies (LBs) and Lewy neurites, intraneuronal and axonal inclusions consisting of aggregated α-synuclein (α-syn), is the pathological hallmark of Parkinson’s Disease (PD) and Dementia with Lewy Bodies (DLB) [1]. This accumulation of α-syn has been associated with impaired functioning of protein degradation mechanisms [2, 3]. One of the main routes for the intracellular degradation of α-syn is autophagy [4, 5], the lysosome-mediated catabolic process in which dysfunctional organelles and proteins are degraded in mammalian cells. Autophagy has a pivotal role in the homeostasis of neurons. Protein quality control and organelle recycling are particularly important in these cells, that have abundant oxidative metabolism and generally do not replicate in adult life. Moreover, reduced function of autophagy was demonstrated to cause intracellular accumulation of proteins and neurodegeneration in in vitro and in vivo experiments [6, 7]. The reliance of neurons on proper functioning of the autophagy-lysosomal pathway (ALP) is supported by observations that the brain is often the most severely affected organ in primary lysosomal storage disorders (LSDs) [8].

After autophagy was implicated in the degradation of α-syn, converging evidence from genetic, pathological and experimental studies has provided indications that the ALP is compromised in PD [9]. In preclinical model systems of PD, the increase of autophagic degradation activity (*autophagic flux*) by rapamycin and lithium, potent autophagy enhancers routinely used in a laboratory setting, resulted in increased clearance of α-syn and neuroprotective effects, which were primarily related to increased autophagy [10]. After these initial findings, similar effects were shown in different preclinical models of PD using other agents that stimulate autophagy, including metformin and trehalose [11]. These studies together suggested a role of autophagy deregulation in the occurrence of different modeled PD-related pathological processes, and suggested that the pharmacological enhancement of autophagy holds promising potential for the development of disease-modifying therapies in PD. However, the selectivity for most of these agents is limited, while upstream ALP components are involved in many other pathways than autophagy. In the context of neurodegenerative disease, in which high-dosage long-term therapies are necessary in order to exert effects in the brain, the broad stimulation of autophagy could therefore result in a wide spectrum of side-effects. Recently, however, several agents that selectively target more downstream ALP components have been developed, which opens up exciting perspectives to combat specific molecular pathogenetic processes relevant for PD.

This study focuses on the emerging role for dysfunctional autophagy in PD, discusses potential molecular targets in the ALP and reviews the evidence obtained in preclinical model systems of PD using autophagy-enhancing strategies, in order to determine the potential of targeted autophagy modulation as disease-modifying therapy in PD.

## Molecular regulation of autophagy

So far, three types of autophagy have been identified based on routes that differ in the way in which substrates eventually reach the lysosomal lumen: microautophagy, chaperone-mediated autophagy (CMA) and macroautophagy. In microautophagy, cytosolic substrates are engulfed directly by the lysosome, after deformation of the lysosomal membrane, in a non-specific way [12]. Macroautophagy is a degradation pathway that involves the formation, elongation and nucleation of double-membrane organelles - called autophagosomes - by which the substrate is sequestered, before fusion with lysosomes [13]. Finally, chaperone-mediated autophagy (CMA) is a highly specific process in which soluble cytosolic proteins containing a KFERQ-related targeting motif are recognized by a chaperone-complex involving the heat-shock cognate protein of 70 kDa (Hsc70). The substrate is subsequently translocated to and internalized by the lysosomal-associated membrane protein 2a (LAMP2a) receptor [14]. Under normal conditions, CMA and macroautophagy occur constitutively at low levels, while these processes are triggered under conditions of cellular stress, including starvation, oxidative stress and presence of protein aggregates [13]. While CMA is characterized by its high specificity, macroautophagy has originally been considered a nonspecific bulk degradation pathway. However, a growing number of selective, specialized types of macroautophagy are described which are generally named to the cargo destined for degradation, for instance the targeted degradation of mitochondria (mitophagy), peroxisomes (pexophagy), the endoplasmic reticulum (ER; reticulophagy), ribosomes (ribophagy), lipid droplets (lipophagy) and many more [15]. These specialized forms of macroautophagy have their own degradation cues and specific ubiquitin-dependent or independent autophagy receptors (reviewed in [16, 17]).

**Fig. 1:**
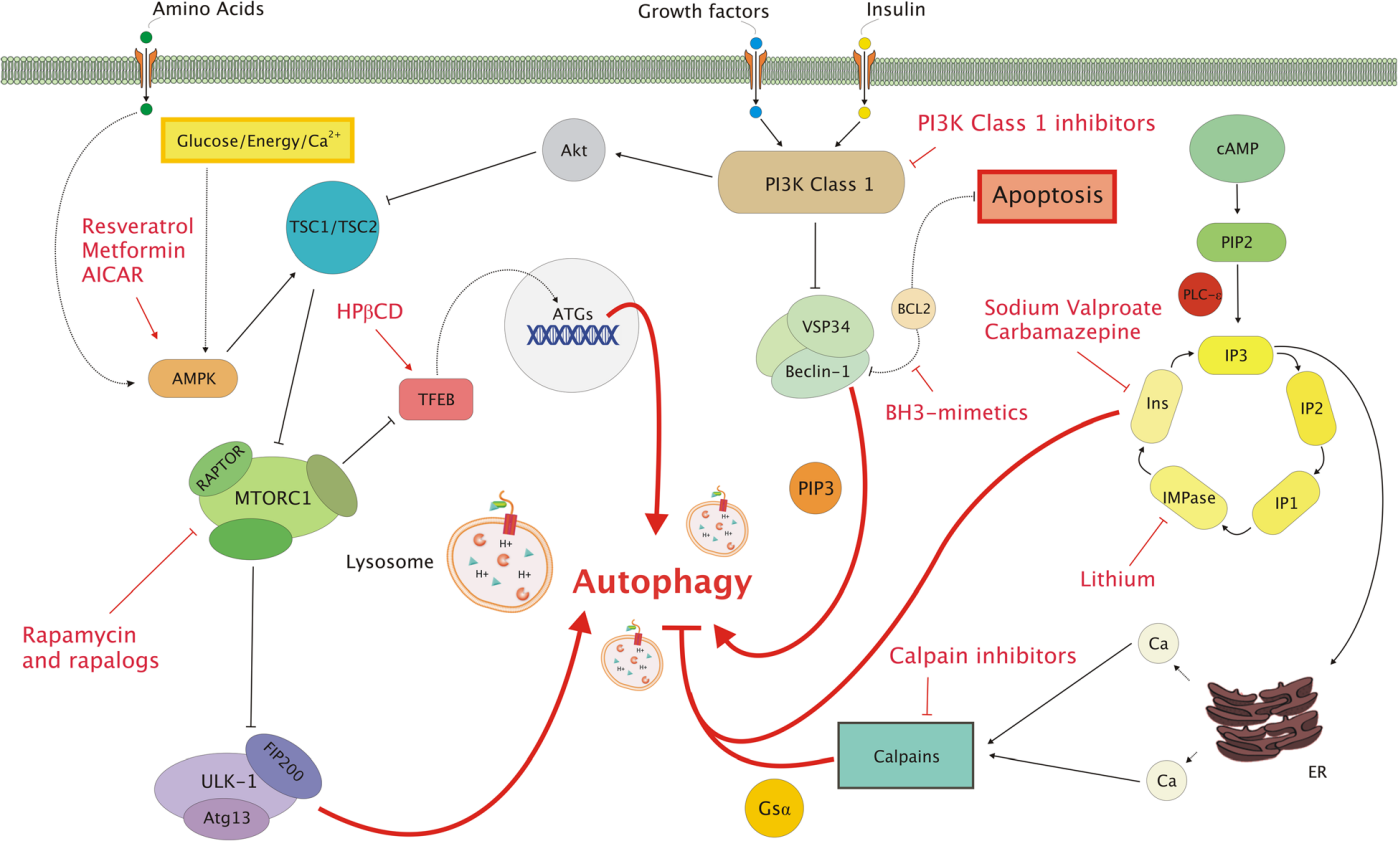
Molecular regulation of macroautophagy and targets for pharmacological stimulation of the autophagy-lysosomal pathway. Situations of amino acid deprivation and low amounts of energy, detected by AMPK, can lead to the inhibition of mTORC1, resulting in the initiation of autophagy via activation of the ULK1-FIP200-Atg13 complex. In this situation, TFEB is dephosphorylated and translocates to the cell nucleus where it binds to ATGs to activate *de novo* gene transcription. Deprivation of growth factors or insulin results in reduced activation of the PI3K Class 1 complex, which promotes the formation of autophagosomes via activation of the Beclin-1-VSP34 complex. A final mTOR-independent pathway, involving the generation of IP_3,_ acts as a negative regulator of autophagy. A number of autophagy-enhancing agents, shown in red, is yet available, allowing to act at different levels of the autophagy-lysosomal pathway

Macroautophagy is an evolutionary highly conserved process and its molecular pathways have been well-characterized after the discovery of autophagy-related genes (*ATG*s) in *Saccharomyces cerevisiae* (reviewed in Bento et al. [18]). The mammalian target of rapamycin (mTOR), a 289-kDa serine/threonine kinase, has been identified as a master regulator of macroautophagy, that can be embedded in two protein complexes: mTORC1 or mTORC2. Macroautophagy is negatively regulated by mTORC1, while mTORC2 is primarily involved in regulation of cellular survival and cytoskeletal organization [19]. Situations of amino acid deprivation relief the direct inhibition of mTORC1 on a protein complex involving UNC-51-like kinase 1 (ULK-1), Atg13 and FIP200, leading to macroautophagy initiation (Fig. 1) [20]. Binding of growth factors or insulin to their corresponding receptors activates the PI3K class 1 protein complex, which can activate mTORC1 via Akt and the tuberous sclerosis complex (TSC1/TSC2 complex) [21]. The activation of PI3K class 1 further results in the inhibition of a macromolecular protein complex including PI3K class 3 (Vps34), Beclin-1 and ATG14L [22], which, when stimulated, promotes autophagosomal membrane nucleation [23]. AMP activated protein kinase (AMPK) detects the intracellular ratio between ATP and AMP and low amounts of energy result in AMPK activation. AMPK activation exerts an inhibiting effect on mTORC1 via the TSC1/TSC2 complex, or in direct phosphorylation of ULK-1, both resulting in the initiation of autophagy [24]. In this way, eukaryotic cells are equipped with a mechanism in which the initiation of autophagy is tightly coupled to cell growth regulation via either inhibition or stimulation of mTOR.

The transcription factor EB (TFEB) has been identified as key regulator of biogenesis and function of lysosomes and functions downstream of mTORC1 [25, 26]. In resting cells, TFEB is localized at the cytosol, where it interacts with mTORC1 and the lysosomal vacuolar-type ATPase complex. The inhibition of mTORC1 activity results in dephosphorylation of TFEB, which then translocates to the cell nucleus and binds to the lysosome-related genes of the CLEAR network, activating a *de novo* gene transcription [27, 28].

In addition to the mTOR-dependent pathways, macroautophagy can be initiated by a pathway working independent of mTOR (Fig. 1) [29]. This pathway, involving Ca^2+^-calpain-G-stimulatory protein α (G_s_α) and cAMP-Epac-PLC-ε-inositol signaling, 1,4,5-triphosphate (IP_3_) acts as a negative mTOR-independent regulator of macroautophagy [30]. The generation of IP3 from PIP2, which is mediated by PLC-ε, results in the release of calcium from the endoplasmatic reticulum (ER). The subsequent activation of calpains leads to cleavage of G_s_α and inhibition of macroautophagy. In addition, the conversion of IP3 induces a chain reaction resulting in the production of Inositol monophosphatase (IMPase) and Inositol (Ins), which also inhibits autophagic processes. In turn, a decrease of IP3 leads to reduced calcium release and AMPK activation, which ultimately results in autophagy induction.

## Converging evidence for a role of ALP dysfunction in PD

### Genetic studies

A substantial amount of recently identified genetic factors has been shown to be involved in or to interact with the ALP, as was reviewed by Gan-Or et al. [31]. Approximately a decade ago, the association between glucosidase beta acid 1 (*GBA1*) mutations and PD was described. Homozygous mutations in the *GBA1* gene, which encodes for the lysosomal hydrolase glucocerebrosidase (GCase), lead to Gaucher Disease (GD), the most common LSD. Although parkinsonism is a rare feature in patients with GD, several GD patients with parkinsonism had relatives with a typical, late-onset form of PD [32]. After confirmation of this observation in large-scale multicenter studies [33] and meta-analyses [34, 35], the presence of pathogenic heterozygous mutations in this gene is now considered as one of the most important risk factors to develop PD. It is estimated that the prevalence of PD patients with GBA mutations is 5–10%, while this percentage can be higher in certain populations [36].

Several other lysosomal genes have been associated with PD [37]. Genome-wide association studies have repeatedly reported an association between scavenger receptor class B member 2 (*SCARB2*) with reduced risk to develop PD [31]. *SCARB2* encodes for the lysosomal integral membrane protein type 2 (LIMP2), which interacts with GCase and is responsible for its transport to the lysosome [38]. The sphingomyelin phosphodiesterase 1 (*SMPD1)* gene encodes for the lysosomal enzyme acid sphingomyelinase (aSMase), which converts sphingomyelin into ceramide. The association of variants of *SMPD1* with increased risk to develop PD has now been repeatedly reported [39–42]. However, *SMPD1* mutations are rare and more studies are needed to determine their significance in PD. Mutations in the *ATP13A2* (*PARK9*) gene, encoding a lysosomal ATPase, cause a rare form of atypical, juvenile-onset autosomal recessive parkinsonism with pyramidal neurodegeneration and dementia - the Kufor-Rakeb syndrome. Finally, some other LSDs have been associated with parkinsonism, including Niemann-Pick type C, Sanfilippo A, GM1 and GM2 gangliosidoses, and Fabry Disease [37]. However, these associations are often based on observations in small patient groups and thus most likely only relevant for very small subpopulations of PD patients.

Apart from mutations in genes that directly encode for lysosomal components, direct and indirect roles have been proposed for numerous other PD-related mutations in the process of autophagy. A rare form of autosomal dominant PD is caused by mutations in the gene encoding vacuolar protein sorting protein-associated protein 35 (*VPS35*), which is involved in endosomal-lysosomal trafficking, a process functionally associated with autophagy [31, 43]. Moreover, numerous autosomal recessive PD genes, including Parkin (*PARK2*), PINK1 (*PARK6*), DJ-1 (*PARK7*) and Fbxo7 (*PARK15*) were found to be implicated in mitophagy, which is the degradation of dysfunctional mitochondria by autophagy [31, 44]. Mutations in the leucine-rich repeat kinase 2 (*LRRK2*) gene are among the most common risk factors for PD together with *GBA1* mutations. Lrrk2 can both be degraded by CMA [45] and macroautophagy [46], but mutated forms of this protein impair CMA function leading to accumulation of substrates, including α-syn [45]. Lrrk2 has also been proposed to have a more general role in autophagy as well, as the accumulation of autophagic vacuoles was observed in a cellular model transfected with mutated *LRRK2* [47]. However, the exact role of Lrrk2 in this process remains elusive [31, 43]. Finally, some proteins encoded by other PD-related genes, including *SNCA* and *MAPT*, were shown to interact with components of the ALP, thereby modifying the functioning of autophagy [31].

Together, genetic studies have demonstrated that numerous genetic risk factors for PD are directly involved in the functioning of the ALP or mitophagy [31], while for other genetic forms of PD, for instance caused by *LRRK2*, *MAPT* and *SNCA* mutations, functional interactions of their gene-products with components of the ALP were demonstrated. However, it is important to note that the prevalence of most of the mutations described here in PD is low, and that they do not necessary lead to PD. The genetic studies further suggest that dysfunctional autophagy in PD can have different underlying gene-deficits.

### Pathological studies

After accumulation of autophagic vacuoles in the postmortem SN of PD patients was first described [48], subsequent postmortem studies demonstrated altered expression of numerous ALP components in the PD brain compared to age-matched controls [49]. Increased levels of microtubule-associated protein 1 light chain 3 (LC3), a marker for autophagosomes, have been observed in the SN of PD patients [50] as well as in the temporal cortex of patients with Dementia with Lewy Bodies [51]. Furthermore, lysosomal depletion was indicated by decreased levels of lysosomal-associated membrane protein type 1 (LAMP1) in the SN of PD patients [50, 52]. Lower levels of the CMA markers LAMP2a and the chaperone Hsc70 have further been observed in various regions of the PD brain compared to controls [53, 54], suggesting CMA deregulation. In addition, the deregulation of lysosomal enzymes, in particular GCase, has been demonstrated in different brain regions [55–57] and CSF [58–60] of PD patients compared to controls, while increased immunoreactivity of the GCase-interactor LIMP2 was observed in dopaminergic neurons in the SN of PD patients [61]. Altered enzyme activities in the PD brain have not only been observed for GCase, but also for cathepsin A and cathepsin D [52, 56]. For cathepsin D, increased [56] and decreased [52] activities were reported in the PD cingulate cortex and SN, respectively, while no changes were found in the frontal cortex [62]. Finally, contradictory results were obtained for P-type ATPase (ATP13A2) in the frontal cortex of PD and DLB patients, for which both decreased [63] and increased [64] protein levels have been reported compared to controls. In the striatum of PD and DLB patients, increased ATP13A2 protein levels were found [64].

A deregulation of the ALP in the PD brain was recently demonstrated by transcriptome studies [65–67]. Alterations for numerous autophagy-related processes, including mTOR signaling, PI3K/AKT signaling and 14-3-3 protein signaling, have been reported in postmortem SN tissue as well as in peripheral blood mononuclear cells (PBMCs) of PD patients [65–67]. The deregulation of the mTOR-dependent pathway in synucleinopathies was underscored by the finding that mTOR protein expression levels were increased in the temporal cortex of patients with DLB in comparison to controls, in particular in neurons displaying α-syn accumulation [68]. Alterations in other upstream autophagy-related proteins were demonstrated in brain tissue of patients with PD and DLB compared to controls [69]. Immunoreactivity of ULK-1, ULK-2, VPS35 and autophagy/Beclin-1 regulator 1 (AMBRA1) was shown within mature LBs [69]. No differences in protein expression levels were observed for these proteins in SN tissue of PD patients, although VPS35 levels were found increased in the temporal cortex of DLB patients [69]. Beclin-1 protein expression levels were found increased in SN tissue [69], but decreased in the cingulate cortex of patients with PD [56]. In the postmortem SN of PD patients, the subcellular localization of TFEB was changed, as TFEB expression in the nuclear department of dopaminergic neurons was significantly decreased in PD patients compared to controls [70]. Moreover, TFEB co-localized with LBs in the same region [70].

In conclusion, pathological studies have shown some evidence for altered ALP protein levels and lysosomal enzyme activity in PD and DLB. However, the reported alterations are generally subtle and sometimes inconsistent, with large overlap between patients and aged controls. Additionally, these pathological studies often also show tremendous variability within the group of PD patients. Together, this may indicate that multiple pathological processes may underpin dysfunctional autophagy in PD or that autophagy is impaired in a subpopulation of PD patients. Moreover, so far, most studies have only focused on the SN of PD patients [49]. As some of the results suggest a differential regulation of autophagy markers between different brain regions, systematic studies that map the distribution of lysosomal and autophagy markers in different stages and molecular subtypes of PD is needed.

### Interactions between autophagy and α-syn

Experimental studies have identified the ALP as key player in the clearance of α-syn. Both the ubiquitin-proteasomal system (UPS) and autophagy were found to be implicated in the clearance of wild-type (WT) α-syn in PC12 cell lines, as the inhibition of both processes results in its accumulation [4]. WT α-syn contains the CMA-targeting motif required for Hsc70 recognition, suggesting that its autophagic degradation is associated with CMA activity. Although its clearance can also be partially mediated by macroautophagy, this system may not be of crucial importance in this process [71]. However, a more important role for macroautophagy has been suggested in situations of increased α-syn burden [37], as macroautophagy has been implicated in the clearance of α-syn oligomers [72], as well as mutant [73] and post-translationally modified forms of α-syn, in cellular [74] and yeast [75] models of PD.

Several studies have further shown the possible involvement of lysosomal enzymes in α-syn degradation. First, genetic or pharmacological reduction of GCase activity results in the accumulation of α-syn in in vitro [76, 77], in mice [77], as well as in a transgenic mouse model of GD [78]. Additionally, the depletion of both GCase and cathepsin D lead to enhanced cell-to-cell transmission of α-syn aggregates in vitro, suggesting a role for these enzymes in the spreading and propagation of synucleinopathy [79, 80]. Together, these studies have shown an intimate relation between autophagy, lysosomal enzymes and α-syn turnover. The verification of macroautophagy and CMA contributions in the turnover of different forms of α-syn in vivo, under physiological and pathological conditions, is ongoing [5].

## Stimulating macroautophagy in preclinical models of PD

Approximately a decade ago, pioneering studies have explored the chemical stimulation of the ALP in PD model systems using rapamycin and lithium, substances that are routinely used to trigger autophagy in vitro. After this, numerous studies have tested the effects of other autophagy-enhancing agents on different modeled PD-related pathological processes. The following section highlights preclinical studies in which effects of chemical agents in preclinical PD models were primarily associated with increased autophagy (summarized in Table 1).

**Table 1:** Reported effects of autophagy-enhancing agents in preclinical PD models

	Target	Agent	Main effect	PD model	Ref
mTOR-dependent pathways	AMPK	Metformin	Reduced cell death	Drosophila melanogaster mutated for LRRK2	[95]
				MPTP mice	[96]
			Reduced phospho-Ser129 α-syn levels	α-Syn overexpressing SH-SY5Y cells	[98]
	AMPK	AICAR	Reduced cell death	LRRK2-mutated Drosophila Melanogaster	[95]
	AMPK (SIRT1)	Resveratrol	Increased α-syn clearance	α-Syn overexpressing PC12 cells	[100]
			Reduced cell death	Rotenone-exposed SH-SY5Y cells	[100, 101]
			Improved mitochondrial functioning	Cultured PARK2-mutant fibroblasts	[102]
	Beclin-1	PREP inhibitor (KYP-2047)	Decreased oligomeric α-syn, increased striatal DA levels	A30P α-syn transgenic mice	[140]
	Beclin-1	Isorhynchophylline	Increased α-syn clearance	N2a cells transfected for WT, A53T, and A30P α-Syn; Embryonic DA neurons	[141]
			Increased α-syn clearance/Reduced α-syn accumulation	WT, A30P, and A53T α-syn expressing PC12 cells	[4]
				B103 neuronal cells expressing α-syn and Beclin-1	[103]
				α-Syn-transgenic mice	[68]
				α-Syn-transgenic rats	[70]
	mTORC1	Rapamycin and Rp analogues (CCI-779, RAD001 and AP23573)	Reduced phospho-Ser129 α-syn levels	α-Syn overexpressing SH-SY5Y cells	[98]
			Reduced cell death	Rotenone-exposed SH-SY5Y cells	[82, 92, 93]
				6-OHDA and MPP+ treated PC12 cells	[83]
				MPTP mice	[50, 83]
				α-Syn-transgenic mice	[68]
				α-Syn-transgenic rats	[70]
				Drosophila melanogaster mutated for PINK-1 and Parkin	[87]
			Improved motor function, reduced synaptic injury	A53T- α-Syn transgenic mice	[84]
			Reduced levodopa-induced dyskinesia	6-OHDA mice; 6-OHDA rats	[85, 86]
			Reduced mitochondrial dysfunction	Rotenone-exposed SH-SY5Y cells	[82, 92]
				Drosophila melanogaster mutated for PINK-1 and Parkin	[87]
	TFEB	2-HPβCD	Increased α-syn clearance	H4 human neuroglioma cells transfected for α-syn	[143]
mTor-independent pathways	IMPase	Lithium	Increased clearance of A53T and A30P α-syn	PC12 cells expressing A53T and A30P α-syn	[88]
			Reduced apoptosis and mitochondrial dysfunction	Rotenone-exposed SH-SY5Y cells	[92, 93]
			Improved motor function, increased viability DA cells in the SN, decreased loss of DOPAC	MPTP mice (combined treatment with lithium and sodium valproate)	[94]
	Ins	Sodium Valproate	Reduced apoptosis and mitochondrial dysfunction	Rotenone-exposed SH-SY5Y cells	[92]
			Improved motor function, increased viability DA cells in the SN, decreased loss of DOPAC	MPTP mice (combined treatment with lithium and sodium valproate)	[94]
	Ins	Carbamazepine	Reduced apoptosis and mitochondrial dysfunction	Rotenone-exposed SH-SY5Y cells	[92]
	SLC2A transporters	Trehalose	Reduced cell loss	Rotenone-treated rats and PC12 cells	[107]
				MPTP mice; A53T α-Syn overexpressing rats	[108, 109]
			Increased α-syn clearance	PC12 cells overexpressing WT and A53T α-Syn	[104, 106]
				NB69 human neuroblastoma cells	[105]
				Rotenone-treated PC12 cells	[107]
				A53T α-Syn overexpressing rats	[109]
			Increased clearance of detergent-insoluble α-syn	A53T α-Syn overexpressing mice	[110]
			Reduced motor deficits	MPTP mice; A53T α-Syn overexpressing rats	[108, 109]
			Reduced neuroinflammation	MPTP mice	[108]
	Unknown	SMERs (SMER 10, 18 & 28)	Increased A53T α-syn clearance	PC12 cells expressing A53T α-syn	[112]
	Unknown	Latrepirdine	Increased α-syn clearance	Saccharomyces cerevisiael, SH-SY5Y cells expressing α-syn and WT mice	[115]
			Decreased cell death	Saccharomyces cerevisiae expressing α-syn	[115]
	Unknown	Spermidine	Reduced motor dysfunction, increased lifespan; Reduced neuronal cell loss	Drosophila melanogaster expressing α-syn; Caenorhabditis elegans expressing α-syn	[118]
	Unknown (polyphenols)	Curcumin	Reduced α-syn accumulation	SH-SY5Y Cells expressing WT and A53T α-syn	[119]
		Kaempferol	Reduced ROS, apoptosis, and mitochondrial dysfunction	Rotenone-exposed SH-SY5Y cells, mouse primary neuronal culture	[120]
		*C. album* polyphenol fractions	Reduced α-syn accumulation; reduced ROS	Hu neuroglioma H4 cells expressing αSyn; Yeast cells expressing αSyn	[121]
	Unknown	Nilotinib	Increased α-syn clearance; improved motor function	Mice expressing A53T α-synuclein; mouse primary cortical neurons	[122, 123]
			Reduced cell death	Mice expressing A53T α-synuclein	[122]
Lysosomes	GCase	Ambroxol	Restoration of lysosomal function; increased GCase activity	GBA1 mutant fibroblasts	[150, 151]
			Reduction of oxidative stress	GBA1 mutant fibroblasts	[150]
	GCase	Isofagomine	Improved motor performance, increased α-syn clearance, reduced neuroinflammation	WT-α-syn overexpressing mice	[159]
	Lysosome	Acidic Nanoparticles	Restoration of lysosomal function; reduced DA cell loss	Cultured ATP13A2 and GBA-mutant fibroblasts; MPTP mice	[145]

*Abbreviations*: *Ref* reference number, *2-HPβCD* 2-Hydroxypropyl-β-cyclodextrin, *α-syn* α-synuclein, *DA* dopaminergic, *DOPAC* 3,4-Dihydroxyphenylacetic acid, *ROS* reactive oxidative stress, *WT* wild-type, *6-OHDA* 6-hydroxydopamine, *MPTP* 1-methyl-4-fenyl-1,2,3,6-tetrahydropyridine, *UPS* ubiquitin-proteasomal system

### Pioneering studies: Rapamycin and lithium

The most thoroughly tested macroautophagy-enhancer is rapamycin, a metabolite isolated from the bacterial strain *Streptomyces hygroscopicus*. The drug inhibits the upstream signaling factor mTOR by binding to its intracellular FKBP12 receptor, thereby disrupting the ability of mTOR to form assemblies with RAPTOR and blocking mTORC1 signaling [10, 81]. Rapamycin was able to reduce α-syn accumulation and blocked α-syn induced neurodegeneration in wild-type, A30P, or A53T α-syn expressing PC12 cells [4] and in α-syn-overexpressing mice [68] and rats [70], respectively. Rapamycin further reduced cell death in cell lines treated with 6-OHDA and rotenone [82, 83], and reduced neuronal death in the methyl-4-phenyl-1,2,3,6 tetrahydropyridine (MPTP) mouse model of PD [50, 83]. In addition, rapamycin improved motor function in A53T α-syn overexpressing mice [84] and relieved L-Dopa-induced dyskinesias in 6-OHDA-treated mice and rats without affecting the therapeutic efficacy of L-Dopa [85, 86]. Finally, rapamycin showed neuroprotective effects in *Drosophila melanogaster* mutated for PINK-1 and Parkin [87]. However, rapamycin interferes in numerous other pathways than autophagy, while prolonged treatment with rapamycin can lead to the inhibition of mTORC2, thereby possibly stimulating other important cellular pathways, for instance influencing cell survival mechanisms. In addition, the use of rapamycin has been associated with a wide spectrum of side-effects, including oral and respiratory infections, stomatitis, leukopenia, hypertriglyceridemia, hypercholesterolemia and immunosuppression [10].

Lithium, which is in use as a mood stabilizer in the treatment of bipolar disorders, is able to induce autophagy in an mTOR-independent manner by direct non-competitive inhibition of IMPase [88]. The neuroprotective effects of lithium in in vivo and in vitro models have repeatedly been reported [89]. However, lithium interferes with many other cellular pathways as well, and numerous mechanisms of action have been proposed for this drug [90]. The use of lithium in bipolar disorders revealed that the drug is associated with a wide spectrum of dose-dependent side-effects, for instance including fine hand tremor, hypothyroidism, hypercholesterolemia, hyperparathyroidism, and hypercalcemia [91]. In preclinical PD models, lithium prevented accumulation of α-syn in PC12 cells expressing A53T and A30P α-syn [88] and protected against rotenone-induced neurotoxicity and cell death via the induction of autophagy in different cell lines [92, 93]. Two other mood-stabilizing agents - sodium valproate and carbamazepine—were demonstrated stimulate autophagy independently from mTOR via Ins, which is downstream of IMPase [92]. Both sodium valproate and carbamazepine were shown to decrease rotenone toxicity and to induce autophagy in SH-SY5Y cells [92]. Moreover, combined treatment of sodium valproate with lithium alleviated motor impairments in MPTP mice, while it protected SN dopaminergic neurons [94]. However, similar to lithium, these agents are non-selective for autophagy and acts on many other cellular pathways as well, possibly resulting in a numerous unwanted effects.

Together, preclinical studies using rapamycin and lithium showed beneficial effects on different modeled PD-related pathological processes, including α-syn aggregation, mitochondrial dysfunction and oxidative stress, which were associated with increased autophagy. Both agents affect many other cellular processes than autophagy, and their side-effect profile makes them unsuited for prolonged high-dosage therapies. However, these pioneering studies have provided important insights into the role of autophagy in PD-related pathological processes, and demonstrated the potential of autophagy-enhancing strategies in experimental settings.

### Other targets for macroautophagy enhancement

#### AMPK

Activation of AMPK, the signaling factor upstream of mTORC1, leads to the inhibition of mTORC1 and initiation of autophagy. Numerous agents are associated with the activation of AMPK, including metformin, 5-aminoimidazole-4-carboxamide ribonucleotide (AICAR) and resveratrol. The administration of metformin, which is used in Diabetes Mellitus, showed neuroprotective effects in in vitro and in two in vivo models of PD: drosophila with mitochondrial dysfunction and MPTP mice [95–97]. Further, metformin decreased Ser-129 phosphorylated α-syn levels in α-syn overexpressing SH-SY5Y cells [98]. Similar results have been reported using AICAR, an drug used in the treatment of acute lymphoblastic leukemia [95–97]. Resveratrol activates AMPK after interaction with its direct target SIRT1 and protective effects of this compound have been reported in different in vitro and in vivo models of PD [99–102], some of which were associated with increased autophagy [100–102].

Trehalose is a disaccharide that inhibits members of the SLC2A (also known as GLUT) family of glucose transporters, leading to an AMPK-dependent - and mTOR-independent - increase in autophagy [103, 104]. The beneficial effects of this drug for cell survival and α-syn clearance have been shown and associated to increased autophagy in different cell lines [104–106] as well as in multiple in vivo models [107–110]. As trehalose is digested by trehalase into glucose in the small intestine, liver and kidney, its bioavailability in the brain may be limited. Recently, two novel trehalase-indigestible and autophagy-inducing disaccharides were developed, which were shown to reduce polyglutamine aggregation in vitro, highlighting the anti-aggregation properties of these agents [111], which, to our knowledge, have not been tested in preclinical models of PD, yet. In sum, AMPK-dependent increase of autophagy showed beneficial effects on different modeled pathological processes in PD. However, importantly, as cellular energy sensor, AMPK is involved in various other key cellular processes, including cell proliferation and survival. Therefore, the global manipulation of its function is likely to induce unwanted effects, and limits its therapeutic potential in the context of PD.

### Other macroautophagy-enhancing agents

A chemical screening approach has resulted in the discovery of three small-molecule enhancers of rapamycin (SMERs): SMER10, SMER18 and SMER28 [112]. These agents induced autophagy and enhanced clearance of mutant A53T α-syn in a PC12 cell line in an mTOR-independent way [112]. Although SMERs have shown promising potential in PD model systems, their specific biochemical pathways remain to be characterized [113]. The antihistamine latrepirdine was shown to enhance autophagy [114] and to attenuate accumulation of α-syn both in vivo and in vitro systems [115]. Latrepirdine entered phase 3 clinical trials for both Alzheimer’s disease (AD) and HD [116], in which was well-tolerated without significant toxicity it failed to show efficacy compared to placebo (more information available on: https://clinicaltrials.gov/). Although latrepirdine was suggested to act preferentially on α-syn [117], it probably intervenes in many cellular processes, while its biochemical pathways are currently unclear [116]. Finally, numerous studies have associated beneficial effects in preclinical models of PD of other compounds, for instance including spermidine [118], different dietary polyphenols other than resveratrol [119–121] and tyrosine kinase inhibitors [122, 123], with increased macroautophagy. However, the exact mechanisms of action for these compounds require further investigation, and these agents may affect many other cellular processes other than autophagy.

## Macroautophagy: a double-edged sword

Together, studies using macroautophagy-enhancing agents in preclinical models of PD have demonstrated that the broad stimulation of autophagy can result in the alleviation of different modeled PD-related pathological processes, such as cell death, α-syn aggregation, oxidative stress and mitochondrial dysfunction (Table 1). However, the clinical translation of these findings is problematic, given that the selectivity of these agents for autophagy is limited, while upstream ALP signaling proteins are involved in many pathways other than autophagy - for instance including apoptosis, cell growth, and immune responses. This lack of selectivity may limit the therapeutic potential of broad autophagy stimulation, as it may result in unwanted effects. Long-term effects and side-effects of broad stimulation of macroautophagy are currently unknown, and more insight in these effects is of crucial importance before application of prolonged high-dose therapies in a clinical setting. In particular, the intimate relation between macroautophagy and apoptosis should be carefully considered. Although macroautophagy and apoptosis comprise two distinct cellular processes, various shared components and mechanisms have been identified by which autophagy and apoptosis regulate each other [124].

Another important danger of broad macroautophagy enhancement is the complex dual role for autophagy in specific contexts. For example, autophagy can have tumor-suppressive, neutral, or tumor-promiting effects in different types and stages of cancer [125]. Although autophagy can act as tumor suppressor, for instance by the removal of damaged organelles, autophagy stimulation can also promote the growth of established cancers in certain contexts [125, 126]. Indeed, excessive stimulation of macroautophagy under specific circumstances has been associated with detrimental effects. First, the observation of a specific type of cell death characterized by extensive autophagic vacuolization of the cytoplasm in PD postmortem brain tissue [48] has led to the highly debated concept of ‘autophagic cell death’. In various in vitro model systems of PD, autophagic cell death was reported in cells treated with excessive levels of dopamine [127], A53T α-syn [128] and oxidative stress [129]. Moreover, treatment with rapamycin in presence of oxidative stress reduced cell viability [129], while the activation of AMPK has been reported to induce cell death in different in vitro models of PD [130].

The activation of macroautophagy has further been associated with the shortening of neurites in a 6-OHDA mouse model, while the indirect activation of mTOR by the inhibition of Akt was able to suppress the retrograde axonal degeneration [131]. Autophagy was further associated with neurite degeneration in LRRK2-mutant SH-SY5Y cells [47] as well as in mouse superior cervical ganglion neurons [132]. Overexpression of both WT and A53T α-syn in SH-SY5Y and PC12 cell lines resulted in a gradual increase in toxicity, mediated by impairment of CMA [133]. The induction of macroautophagy in these models led to neuronal death, indicating that a compensatory upregulation of macroautophagy under the circumstances of increased α-syn burden can have detrimental effects [133]. Furthermore, the activation of macroautophagy in primary cortical neurons overexpressing A53T α-syn caused mitochondrial destruction and loss, as well as neuronal degeneration [134]. Finally, another form of autophagy-dependent cell death – autosis - was recently described, although its exact mechanisms remain unclear [135].

Taken together, these experimental findings demonstrated that activation or stimulation of macroautophagy can have detrimental effects under specific circumstances. Maintaining the balance between protective and detrimental effects is therefore of vital importance in therapeutic approaches to stimulate broad autophagy [136], which implies extensive knowledge about dosage and timing for such therapies and may limit their current therapeutic applicability.

## Selective targeting of ALP components

### Beclin-1

The activation of Beclin-1 leads to autophagosome formation and initiation of autophagy, independent from mTOR (Fig. 1). Gene-transfer induced overexpression of Beclin-1 was able to reduce accumulation of α-syn in α-syn overexpressing mice [137] and PC12 cells [138], suggesting that the Beclin-1 pathway may be a viable therapeutic target in PD. Although numerous drugs have been associated with increased Beclin-1 activity, including tamoxifen, Beclin-1 expression mimetics (BH3 mimetics) and the peptide Tat-Beclin-1, only few studies tested pharmacological Beclin-1 stimulation in PD models [139–141]. One study showed protective effects of tamoxifen, a drug widely used in the treatment of breast cancer, in a MPP^+^ rat model, in which it suppressed radical generation [139]. However, this effect was not attributed to increased autophagy. Another study reported that the inhibition of prolyloligopeptidase (PREP) resulted in a Beclin-1-dependent increase of autophagy together with a reduction of α-syn aggregates in cell models and α-syn transgenic mice [140]. Finally, isorhynchophylline, a natural alkaloid, was reported to promote the clearance of WT, A53T and A30P α-syn monomers, as well as oligomeric and aggregated forms of α-syn in neuronal cell lines, in which its autophagy-enhancing activity was shown to be Beclin-1-dependent [141]. As the BH-3 domain of Beclin-1 interacts with the pro-survival BCL-2 family members, it was expected that binding of Beclin-1 to BCL2 would induce apoptosis, potentially resulting in undesired side-effects [126]. However, overexpression of Beclin-1 does not result in induction or enhancement of apoptotic processes, which suggested that the functional interaction of Beclin-1 and BCL-2 is unidirectional, resulting in inhibitory effects on autophagy without modifying apoptotic processes [126].

### Macroautophagy stimulation downstream of mTOR

**Fig. 2:**
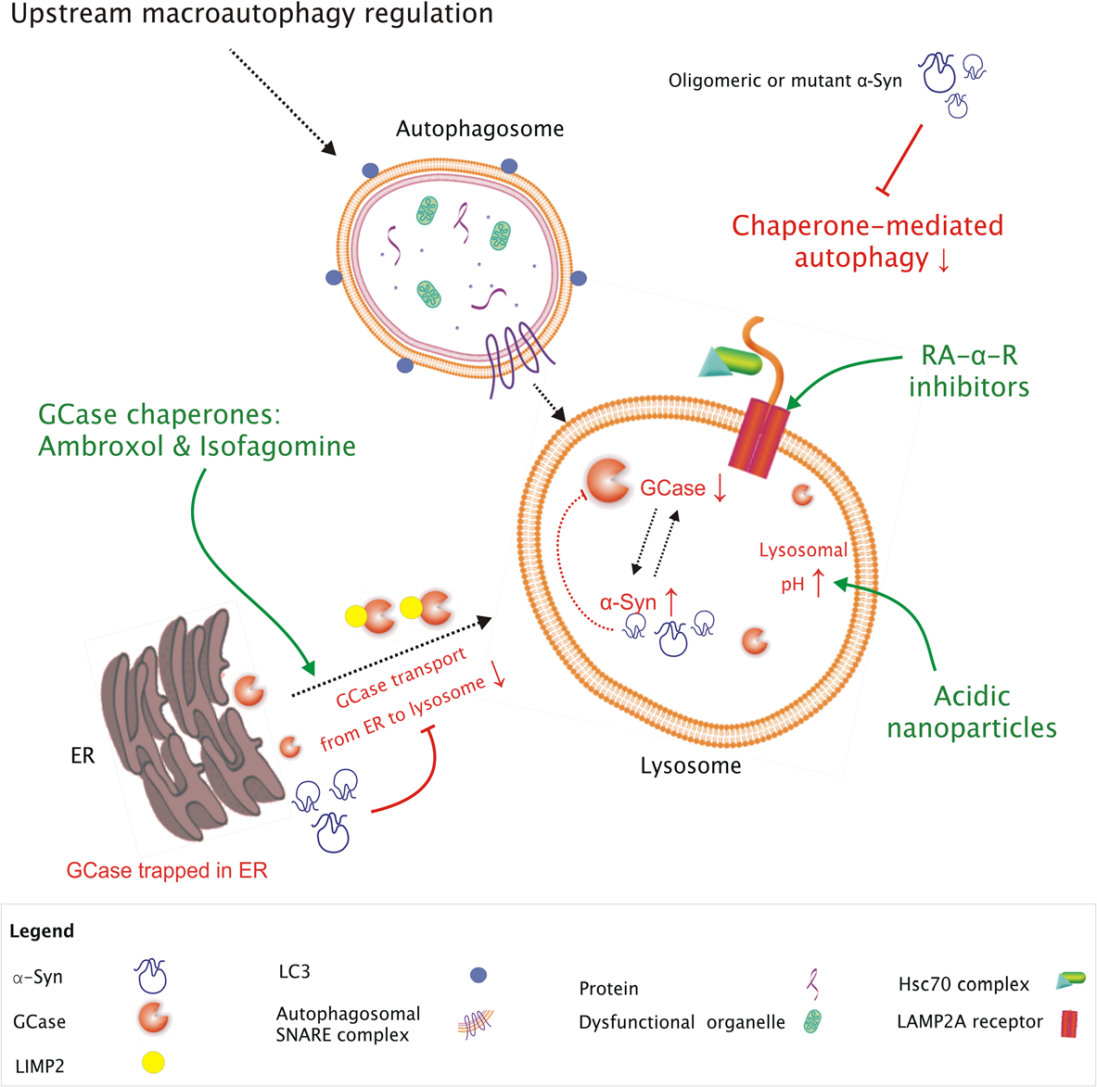
Strategies to combat α-synuclein aggregation by the downstream enhancement of autophagy. Oligomeric and mutant forms of α-synuclein impair CMA functioning in PD, which may be alleviated by the recently developed retinoic acid alpha receptor inhibitors (RA-α-R inhibitors). The intralysosomal presence of α-synuclein further results in impaired GCase functioning, while it blocks the transport of GCase from the ER to the lysosome. Together, these processes lead to reduced GCase hydrolase activity and lysosomal dysfunction in PD. Small-molecule GCase chaperones, including ambroxol and isofagomine, specifically target the misfolded GCase trapped in the ER to increase trafficking of GCase to lysosomes. Acidic nanoparticles improve lysosomal functioning by lowering the pH within the lysosomal lumen

As the main transcriptional regulator of the CLEAR network, which coordinates biogenesis and function of lysosomes, TFEB is a crucial link between the upstream signaling pathways that regulate macroautophagy and lysosomes [26]. Therefore, TFEB forms an attractive target to stimulate macroautophagy downstream of mTOR, by intervening at the transcriptional level (Fig. 2). The first approaches to stimulate TFEB in PD models have yielded promising results. Overexpression of TFEB was demonstrated to eliminate α-syn oligomers and to rescue midbrain dopamine neurons from α-syn toxicity in α-syn overexpressing rats [70]. In addition, delayed activation of TFEB via inhibition of mTORC1, achieved using a derivate of rapamycin (CCI-779), alleviated α-syn pathology and was associated with increased autophagic markers and the nuclear translocation of TFEB [70]. 2-Hydroxypropyl-β-cyclodextrin enables the chemical activation of TFEB [142], and was recently shown to promote autophagic clearance of α-syn [143]. These findings provided a mechanistic link between TFEB function and α-syn aggregation, highlighting the role of TFEB in synucleinopathy and the potential of TFEB-activating strategies to combat α-syn aggregation in PD.

### Direct targeting of lysosomes

The modulation of lysosomes has recently emerged as an attractive strategy to selectively stimulate the ALP in PD. Recent work showed that acidic nanoparticles were able to stimulate lysosomal degradation by the lowering of lysosomal pH [144], reverting lysosomal dysfunction in three different PD genetic models, including ATP13A2-mutant cells and GBA-mutant cells [145]. Moreover, acidic nanoparticles attenuated dopaminergic cell death in MPTP-treated mice, providing evidence for the feasibility of such strategies in vivo [145]. These findings highlight the potential of pharmacological interventions on the lysosomal system.

Another interesting approach is the direct targeting of specific lysosomal enzymes such as GCase to stimulate the intralysosomal degradation of α-syn. Restoration of GCase expression by an adeno-associated virus reduced α-syn aggregation and, interestingly, its expression in the hippocampus was able to reverse cognitive impairment in a GD mouse model [78, 146, 147]. As results from the enzyme-replacement therapies in GD show that GCase does not cross the blood-brain barrier (BBB), the development of small-molecule chaperones to correct the folding of GCase - thereby enhancing GCase activity and lysosomal function - is currently a topic of great interest [148]. Small-molecule chaperones specifically target the misfolded GCase trapped in the ER, stabilizing the active form of the enzyme and increases trafficking of GCase to lysosomes (Fig. 2) [149].

The pharmacological chaperone ambroxol, which has been in use for airway diseases, was shown to increase GCase activity and reduce oxidative stress in PD fibroblasts carrying GBA1 mutations [150]. Furthermore, ambroxol was shown to restore Cathepsin D, LIMP2 and Saposin C levels [151], which are essential for the proper functioning of GCase [149, 152]. In this study, ambroxol acted preferentially on lysosomal dynamics, as it did not interfere with Parkin levels or proteasome 20S activity [151]. Although ambroxol was initially expected not to have CNS effects at clinically used doses [153], the drug was reported to cross the BBB in mice [154]. Recently, a pilot study carried out in neuronopathic GD patients showed that high-dose oral ambroxol could cross the BBB, being also well-tolerated, in addition to enzyme-replacement therapy. In these patients, ambroxol increased lymphocyte GCase activity, reduced CSF glucosylsphingosine levels and was associated with improvements in myoclonus, seizures and pupillary light reflex dysfunction. Accordingly, ambroxol represents an interesting candidate waiting for clinical trials in PD. Other small-molecule chaperones such as AT2101 (isofagomine) as well as histone deacetylase inhibitors have been reported to increase GCase levels in GD mouse models and fibroblasts [155–158], and are currently tested in the context of PD. Additionally, the administration of isofagomine to human WT-α-syn overexpressing mice improved motor performance, reduced α-syn immunoreactivity in nigral dopaminergic neurons and reduced inflammatory microglial activation [159].

Together, small chaperone activity-enhancers of GCase were demonstrated to improve lysosomal function and enhance α-syn clearance in preclinical models of PD. However, a disadvantage of these chaperones is that they inhibit GCase activity by binding the catalytic site of the enzyme, suggesting that the balance between chaperone functions and inhibitory activity should be carefully considered when using these compounds [149]. Therefore, molecular chaperones that do not bind the catalytic site of GCase are required to exert maximal effects. Recently, a high throughput screening has led to the identification of pyrazolopyrimidine derivatives that did not inhibit GCase, but still facilitated its translocation to the lysosome [160]. These agents were able to enhance GCase activity, reduce substrate accumulation and normalize oxygen species production in a macrophage model of GD [161]. Another recent study identified a small-molecule noninhibitory chaperone of GCase (NCGC607) by high-throughput screening [162]. This chaperone was demonstrated to restore GCase activity and protein levels and reduced substrate levels, while it reduced α-syn levels in iPSC-derived dopaminergic neurons [162]. This study underscores the potential of small-molecules targeting GCase activity and protein to prevent or reduce accumulation of α-syn levels in vitro.

## Targeting chaperone-mediated autophagy

CMA impairment in PD has been demonstrated by the lower levels of the CMA markers LAMP2a and the chaperone Hsc70 in various regions of the PD brain compared to controls [53, 54], while a central role for this system is proposed in the degradation of WT α-syn [5]. An attractive alternative to the stimulation of macroautophagy may therefore be the downstream targeting of CMA components, at the level of LAMP2a and Hsc70. The overexpression of LAMP2a has been induced in human SH-SY5Y cells, rat primary cortical neurons in vitro and nigral dopaminergic neurons in vivo [163]. In these different models, overexpression of LAMP2a decreased α-syn accumulation and protected against the α-syn-induced dopaminergic degeneration [163]. Interestingly, retinoic acid alpha receptors (RA-α-Rs) were recently identified as CMA inhibitors and synthetic derivatives of all-trans-retinoic acid were developed in order to neutralize this effect [164]. These derivatives were able to specifically stimulate CMA without a compensatory blockage of macroautophagy, while LAMP2a was identified as one of their downstream targets [164]. However, so far, no studies have reported the chemical modulation of CMA in preclinical models of PD yet.

## Modulation of mitophagy

The involvement of different PD-related genes in mitophagy suggest a role for impaired mitochondrial clearance in PD. By stimulating the removal of damaged mitochondria, the therapeutic stimulation of mitophagy may therefore exert neuroprotective effects. However, pharmaceutical agents that selectively increase mitophagy are currently lacking. The compounds used in vitro to trigger mitophagy, such as trifluorocarbonylcyanide phenylhydrazone (FCCP) and antimycin/oligomycin combinations, are toxic and therefore not considered for therapy [165]. The p62-mediated mitophagy inducer (PMI) may be a promising chemical candidate, as it is suggested to enhance endogenous levels of mitophagy [166]. However, no effects of this agent in preclinical models of PD have been described yet. More insights into the complex dynamics of mitochondria and the identification of targets able to specifically stimulate mitochondrial clearance may allow the development of innovative neuroprotective therapies for PD and other neurodegenerative disorders.

## Autophagy-related micro-RNAs

In recent years, an important role has been suggested for micro-RNAs (miRs) in the post-translational regulation of autophagy at various stages of the ALP [167], which interconnect autophagy with other cellular signaling systems. A recent study showed the down-regulation of miR-124, which is predicted to regulate 52 genes of the ALP in a MPTP mouse model [168, 169], while the delivery of an miR-124 agomir attenuated lysosomal depletion and cell death [169]. A different study demonstrated an important role for different miRs in the regulation of CMA markers LAMP2a and Hsc70 [170]. Interestingly, transfection of these miRs resulted in decreased LAMP2a and Hsc70 protein levels as well as in significant α-syn aggregation [170]. Moreover, CMA-related miRNAs were significantly increased in SN and amygdala of PD patients [170]. A better understanding of the interaction between miRs and ALP components and their role in PD may contribute to valuable insights in autophagy regulation and the cross-talk of autophagy with other cellular processes, and possibly to future autophagy-enhancing strategies [169, 170].

## The therapeutic value of autophagy enhancers in PD: considerations and perspectives

The identification of autophagy as key player in the degradation of α-syn has suggested the potential of autophagy-enhancing strategies as disease-modifying therapy to combat α-syn aggregation in PD. Indeed, stimulation of autophagy by known autophagy enhancers such as rapamycin and lithium, resulted in increased α-syn clearance in different α-syn overexpressing preclinical model systems of PD. Moreover, interestingly, increased autophagy levels were associated with neuroprotective effects in toxic model systems of PD, as pharmacological autophagy enhancement ameliorated cell death and mitochondrial dysfunction, possibly mediated by the increased clearance of damaged mitochondria (Table 1). Together, pioneering studies have highlighted potential effects of autophagy-enhancement on different modeled PD-related pathological processes. However, selectivity of the early autophagy-enhancing agents and their targets is limited and broad macroautophagy stimulation may result in a wide spectrum of dose-dependent side-effects, making it less suited for high-dosage long-term therapies. In addition, the broad and excessive stimulation of macroautophagy under specific, yet not fully characterized, circumstances can have detrimental effects, including autophagic cell death.

The recent development of agents selectively targeting ALP core components - including TFEB, lysosomes and GCase—as well as CMA factors such as LAMP2a, enables more specific targeting of pathogenetic processes, and may harbor more perspective for the development of disease-modifying therapies in PD than the broad activation of macroautophagy. CMA is a highly specific process that is increasingly recognized as a key system in the degradation of WT α-syn [37], which highlights the demand for the discovery and development of chemical substances able to specifically stimulate CMA traffic. Targeting lysosomes or GCase by acidic nanoparticles and small-molecule chaperones, respectively, has emerged as attractive strategy to encounter lysosomal depletion in PD, representing an area of intensive investigation. Moreover, the modulation of downstream ALP components and CMA could lead to more controlled activation of macroautophagy, as substantial cross-talk takes place between CMA and macroautophagy [37, 171]. In the study by McNeill et al., for instance, the GCase chaperone ambroxol exerted marked effects on TFEB expression, which suggested that the protective effects were partially mediated by increased macroautophagy [150]. The identification of the exact mechanisms underlying the cross-talk between autophagy pathways, lysosomes and GCase may allow to identify potential dangers and to determine optimal efficacy of downstream autophagy-enhancing approaches.

Establishing the patient selection, optimal dosage, and timing for maximal therapeutic efficiency will be crucial next steps in the design of autophagy-enhancing therapies. Timing of the autophagy-enhancing therapies may be of particular importance, given the hypothesized differential roles for proteolytic systems in α-syn degradation in different stages of aggregation [37]. In vitro experiments suggested that a compensatory upregulation of macroautophagy under the circumstances of increased α-syn burden can have detrimental effects [133]. LB-like aggregates were further shown to impair macroautophagy by blocking clearance of autophagosomes [172], indicating that excessive macroautophagy stimulation in situations of advanced synucleinopathy may result in autophagic cell death. A better understanding of the role of ALP dysfunction in different stages of PD is therefore essential for the design of future disease-modifying therapies.

Pathological and genetic studies further suggested that various molecular and genetic deficits can underlie dysfunctional autophagy in PD. Therefore, the nonspecific stimulation of autophagy may have differential outcomes depending on the ﻿molecular subtype ﻿of PD. For instance, upregulation of autophagy in the context of lysosomal dysfunction, in which lysosomal clearance of autophagosomes is impaired, may lead to further accumulation of autophagosomes. More insight into the contribution of dysfunctional autophagy to the progression of these different genetic and molecular subtypes of PD is of key importance in order to identify potential dangers and to develop autophagy-enhancing therapies that target subtype-specific key pathological processes. The development of relevant model systems for these subtypes of PD is therefore urgently required. Finally, to monitor therapeutic response of autophagy-enhancing agents, the development of novel assays that reliably measure dynamics in autophagic flux in vivo are highly desired [11].

## Conclusion

In conclusion, pioneering studies using macroautophagy-enhancing agents in preclinical models have provided important knowledge about the role of autophagy in PD and highlighted the promising perspective of autophagy enhancement in this disease. The neuroprotective actions of broad autophagy enhancement were demonstrated in various in vivo and in vitro models, with effects on various modeled neuropathological processes. However, the therapeutic potential of macroautophagy-enhancing agents may be limited due to their lack of selectivity and the double-edged sword properties of macroautophagy. Recently developed compounds that selectively target downstream components of the ALP, including GCase, TFEB and CMA elements, exert more specific effects on autophagy and may have exciting therapeutic perspective. Although several fundamental questions need to be further addressed before these novel agents can be applied in a clinical setting, the research field of autophagy is developing quickly and clinically relevant updates on these topics may be expected soon. Further characterization of dysfunctional autophagy in different stages as well as genetic and molecular subtypes of PD in combination with the effective clinical translation of downstream autophagy regulation offers exciting new avenues for the development of therapeutic strategies in PD.

## Abbreviations

6-OHDA: 6-Hydroxydopamine
AICAR: Aminoimidazole-4-carboxamide ribonucleotide
ALP: Autophagy-lysosomal pathway
AMBRA1: Autophagy/Beclin-1 regulator 1
AMPK: AMP activated protein kinase
aSMase: Acid sphingomyelinase
ATP13A2: P-type ATPase
BH3 mimetics: Beclin-1 expression mimetics
CMA: Chaperone-mediated autophagy
DLB: Dementia with Lewy Bodies
ER: Endoplasmic reticulum
FCCP: Trifluorocarbonylcyanide phenylhydrazone
GBA1: Glucosidase beta acid 1
GCase: Glucocerebrosidase
GD: Gaucher Disease
G_s_α: Ca^2+^-calpain-G-stimulatory protein α
Hsc70: Heat-shock cognate protein of 70 kDa
IMPase: Inositol monophosphatase
Ins: Inositol
IP3: 1,4,5-Triphosphate (IP_3_)
LAMP: Lysosomal-associated membrane protein
LB: Lewy Bodies
LC3: Microtubule-associated protein 1 light chain 3
LIMP2: Lysosomal integral membrane protein type 2
*LRRK2*: Leucine-rich repeat kinase 2
LSD: Lysosomal storage disorder
miR: micro-RNA
MPTP: Methyl-4-phenyl-1,2,3,6 tetrahydropyridine
mTOR: Mammalian target of rapamycin
PD: Parkinson’s Disease
PMI: p62-Mediated mitophagy inducer
PREP: Prolyloligopeptidase
*SCARB2*: Scavenger receptor class B member 2
SMER: Small-molecule enhancer of rapamycin
*SMPD1*: Sphingomyelin phosphodiesterase 1
TFEB: Transcription factor EB
TSC1/TSC2 complex: Tuberous sclerosis complex
ULK-1: UNC-51-like kinase 1
UPS: Ubiquitin-proteasomal system
*VPS35*: Vacuolar protein sorting protein-associated protein 35
WT: Wild-type
α-syn: α-synuclein
